# Immune protection is dependent on the gut microbiome in a lethal mouse gammaherpesviral infection

**DOI:** 10.1038/s41598-020-59269-9

**Published:** 2020-02-11

**Authors:** Jordan R. Yaron, Sriram Ambadapadi, Liqiang Zhang, Ramani N. Chavan, Scott A. Tibbetts, Shahar Keinan, Arvind Varsani, Juan Maldonado, Simona Kraberger, Amanda M. Tafoya, Whitney L. Bullard, Jacquelyn Kilbourne, Alison Stern-Harbutte, Rosa Krajmalnik-Brown, Barbara H. Munk, Erling O. Koppang, Efrem S. Lim, Alexandra R. Lucas

**Affiliations:** 1 Center for Personalized Diagnostics, The Biodesign Institute, Arizona State University, Tempe, Arizona USA; 2 Center for Immunotherapy, Vaccines and Virotherapy, The Biodesign Institute, Arizona State University, Tempe, Arizona USA; 3 Center for Fundamental and Applied Microbiomics, The Biodesign Institute, Arizona State University, Tempe, Arizona USA; 4 Department of Molecular Genetics & Microbiology, College of Medicine, University of Florida, Gainesville, Florida USA; 5 Cloud Pharmaceuticals, Research Triangle Park (RTP), North Carolina USA; 6 School of Life Sciences, Arizona State University, Tempe, Arizona USA; 7 Center of Evolution and Medicine Arizona State University, Tempe, Arizona USA; 8 Structural Biology Research Unit, Department of Integrative Biomedical Sciences, University of Cape Town, Rondebosch, Cape Town South Africa; 9 KED Genomics Core, Arizona State University, Tempe, Arizona USA; 10 Swette Center for Environmental Biotechnology, The Biodesign Institute, Arizona State University, Tempe, Arizona USA; 11 School of Sustainable Engineering and the Built Environment, Arizona State University, Tempe, Arizona USA; 12 Department of Basic Sciences and Aquatic Medicine, Faculty of Veterinary Medicine, Norwegian University of Life Sciences, Oslo, Norway

**Keywords:** Recombinant protein therapy, Microbiome, Virology

## Abstract

Immunopathogenesis in systemic viral infections can induce a septic state with leaky capillary syndrome, disseminated coagulopathy, and high mortality with limited treatment options. Murine gammaherpesvirus-68 (MHV-68) intraperitoneal infection is a gammaherpesvirus model for producing severe vasculitis, colitis and lethal hemorrhagic pneumonia in interferon gamma receptor-deficient (IFNγR^−/−^) mice. In prior work, treatment with myxomavirus-derived Serp-1 or a derivative peptide S-7 (G_305_TTASSDTAITLIPR_319_) induced immune protection, reduced disease severity and improved survival after MHV-68 infection. Here, we investigate the gut bacterial microbiome in MHV-68 infection. Antibiotic suppression markedly accelerated MHV-68 pathology causing pulmonary consolidation and hemorrhage, increased mortality and specific modification of gut microbiota. Serp-1 and S-7 reduced pulmonary pathology and detectable MHV-68 with increased CD3 and CD8 cells. Treatment efficacy was lost after antibiotic treatments with associated specific changes in the gut bacterial microbiota. In summary, transkingdom host-virus-microbiome interactions in gammaherpesvirus infection influences gammaherpesviral infection severity and reduces immune modulating therapeutic efficacy.

## Introduction

Viral infections induce potent immune responses, an immunopathogenesis that can lead to severe complications with sepsis or leaky capillary syndromes and very high mortality and limited effective treatments, a true unmet clinical need. Sepsis has an associated risk of disseminated intravascular coagulation (DIC) with thrombosis, hemorrhage and shock^1–3^. One such group of viruses with known severe complications are the gammaherpesviruses (GHV). The murine gammaherpesvirus-68 (MHV-68) is a widely used, well-controlled laboratory model of GHV host-pathogen interaction with genetic similarity to the human viruses Epstein-Barr virus (EBV) and Kaposi’s sarcoma-associated herpesvirus (KSHV)^4^.

Inflammatory vasculitic syndromes (IVS) are a group of rare, heterogeneous and devastating inflammatory conditions of the body’s extensive system of blood vessels with increased morbidity, including sudden loss of vision, aneurysm, aortic arch syndrome, stroke, and associated increases in mortality^5–7^. The etiology of many systemic vasculitides is currently unknown, with proposed mechanisms ranging from induction by fungal spores to herpesviruses infections (e.g., zoster)^7,8^. A lethal IVS large vessel arteritis model which closely mimics human Giant Cell Arteritis, Kawasaki’s disease and Takayasu’s arteritis can be induced by high dose intraperitoneal MHV-68 infection of interferon gamma receptor knockout (IFNγR^−/−^) mice^9^. Many infected mice display extensive pulmonary hemorrhage and consolidation, mimicking DIC in viral sepsis, which has a very high attendant mortality^10^. A significant number of mice also have marked colon dilatation reminiscent of toxic megacolon^11^, in addition to aggressive inflammatory cell invasion with hemorrhagic lung consolidation. Herpesvirus infections have been associated with inflammatory bowel diseases^12–14^ and gastrointestinal involvement in systemic vasculitides has been reported by other groups^15,16^. As other gastrointestinal inflammatory conditions are associated with, or driven by, microbiome changes, the gut microbiome may have a role in the pathogenesis of GHV and specifically MHV-68 infections.

We have previously reported that immune modulating proteins from myxomavirus (MYXV) provide a new class of anti-inflammatory treatments when delivered as recombinant protein or by ectopic expression after Adeno-associated virus (AAV) delivery^17–20^. MYXV is the causative agent of lethal myxomatosis in the European rabbit (*Oryctolagus cuniculus*), expressing highly potent immune evasion proteins that act as virulence factors in MYXV infections. MYXV is not a pathogen for mice or humans. When purified as isolated recombinant proteins, these immune modulating biologics can modify disease progression in a wide range of inflammatory diseases in preclinical animals and man^21^. In prior work, the MYXV-derived serine protease inhibitor (serpin) Serp-1 and a Serp-1 reactive center loop (RCL) peptide S-7 (G_305_TTASSDTAITLIPR_319_) significantly improved survival, reducing pulmonary and aortic inflammation as well as hemorrhagic lung consolidation after MHV-68 infection^10,22^. Serp-1 targets the coagulation pathways, both thrombolytic and thrombotic, and has proven, highly effective anti-inflammatory functions^23,24^. Antibiotic treatment of IFNγR^−/−^ mice prior to MHV-68 infection abrogated the efficacy of S-7 treatment for lethal MHV-68 infections, leading to significantly reduced survival. Therapeutically modified S-7 peptides (MPS7–8 and MPS7–9), designed based upon the serpin crystal structure, maintained efficacy in this model with improved survival after MHV-68 infection^25^. The MPS7 peptides have predicted increased hydrogen bonds when compared to the native S-7 peptide^25^.

The pathophysiologic role of the gut bacterial microbiome in gammaherpesviral infections and on immune modulating treatments has not previously been investigated. We report here a systematic examination of the role of the gut bacterial microbiome in MHV-68 infection and on Serp-1 and Serp-1-derived S-7 peptide treatment in MHV-68 infections.

## Results

### Serp-1 protein and S-7 peptide treatments improve survival after MHV-68 infection in IFNγR^−/−^ mice

**Figure 1:**
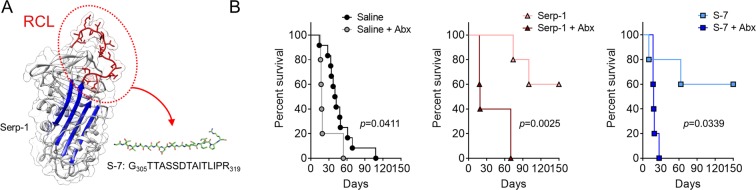
Serp-1 and Serp-1 RCL-derived peptide S-7 improve survival in lethal MHV-68 infection. (**A**) Structure of Serp-1 with A-beta sheet (blue) and reactive center loop (RCL; red) highlighted. RCL-derived peptide S-7 illustrated in green. (**B**) Kaplan-Meier curve depicting survival of IFNγR^−/−^ mice infected with MHV-68 and treated with saline (left; N = 12 and 5), Serp-1 (middle; N = 5 and 5), or S-7 (right; N = 5 and 5) without or with antibiotics.

MHV-68 infections establish latency with chronic infection in wildtype mice^26^, causing only flu-like symptoms. Conversely, intraperitoneal infection of mice with underlying immune deficiency, as for IFNγR^−/−^ mice, results in a lethal large vessel vasculitis, colitis and hemorrhagic pneumonia with early mortality^9,27^. Intranasal infection, an alternative model for MHV-68, is not a model for severe vasculitic syndromes. Our group and others have used this intraperitoneal infection model of vasculitis to study the pathogenesis of gammaherpesvirus host-pathogen interactions, as well as to investigate the potential for anti-inflammatory treatment of associated inflammatory vasculitic syndromes^10,11,22,25,28,29^. Intraperitoneal infection of IFNγR^−/−^ mice with 12.5 × 10^6^ pfu was performed with follow-up for 150 days to determine survival rates. MHV-68 infection with administration of control saline treatment alone for 30 days was lethal, with a median survival of 41 days. Serp-1 (100 ng/g) or S-7 (100 ng/g) intraperitoneal (IP) injections for 30 days improved survival to 60% at 150 days (*p* = 0.0022 and *p* = 0.0218, respectively) (Fig. 1B). Thus, Serp-1 and S-7 protect IFNγR^−/−^ mice after lethal MHV-68 infection^10,22^.

### Loss of gut bacteria accelerates MHV-68 induced disease

**Figure 2:**
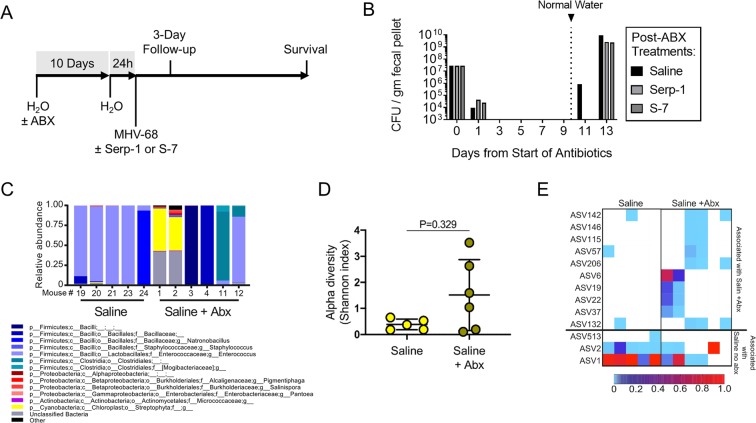
Antibiotic treatment accelerates MHV-68 lethality and alters the gut bacterial microbiome. (**A**) Overview of experimental design. Mice are treated with a broad-spectrum antibiotic cocktail in their drinking water for 10 days, placed on normal water for 1 day, infected with MHV-68 with or without treatments with follow-up for survival. (**B**) Antibiotic-medicated water completely ablates bacterial microbiome contents during the course of the 10-day pre-treatment, which recovers by three days after removal of antibiotics. (**C**) 16 S microbiome relative abundance (genus level) and (**D**) Bacterial alpha diversity (Shannon Index) is shown for saline-treated mice with and without antibiotics. (**E**) Heatmap showing ASV abundance in mice treated with saline in the presence or absence of antibiotic pre-treatment.

We hypothesized that the gut bacterial microbiome may play a role in the host-pathogen interactions of MHV-68 induced disease through transkingdom interactions. We completed a systematic analysis of the effect of microbiome depletion on MHV-68 induced disease progression (Fig. 2A). Mice were maintained on medicated water containing a broad-spectrum cocktail of four antibiotics: bacitracin, gentamicin, streptomycin and ciprofloxacin (Table 1) in order to suppress gut bacterial populations. During this time the density of the gut bacteria (as measured by colony forming units [CFU] per gram fecal pellet) was markedly reduced, validating a decrease in bacterial load (Fig. 2B). After ten days, mice were returned to normal drinking water for 24 hours, during which time the antibiotic treatment maintained suppression of the microbiome (Fig. 2B). Mice were subsequently infected by IP injection of MHV-68 and injected with control saline daily for 30 days and survival assessed for up to 150 days (Fig. 1B).

**Table 1:** Antibiotic cocktail components.

Antibiotic	Dose	Target class	Mechanism of action
Bacitracin	1 g/L	Gram-positive	Cell wall synthesis inhibition^88^
Gentamicin	0.5 g/L	Gram-negative and Gram-positive	Ribosome inhibition^89^
Streptomycin	2 g/L	Gram-negative and Gram-positive	Ribosome inhibition^90^
Ciprofloxacin	0.125 g/L	Gram-negative and Gram-positive	DNA replication inhibition^91^

Suppression of gut bacteria by antibiotic treatment markedly accelerated the course of MHV-68 infection. Saline-treated mice exhibited a median survival of 41 days, while antibiotic-treated mice had a median survival of 19 days (p = 0.0411; Fig. 1B). 16 S rRNA gene amplicon sequencing was subsequently performed on DNA extracts from the large intestines of individual mice at 3 days post- MHV-68 infection in order to assess early changes in the gut microbiota. We detected a distinct dysbiosis in mice pre-treated with antibiotics prior to MHV-68 infection versus mice with a stable bacterial microbiome prior to infection (Fig. 2C). This dysbiosis was associated with a non-statistically significant trend towards increased diversity in amplicon sequence variant (ASV) richness (Fig. 2D; p = 0.329). Sub-population analysis revealed significant changes in candidate ASVs in MHV-68 infected mice that were associated with antibiotic treatment (Fig. 2E, top panels; increased abundance after antibiotics) or with no antibiotic treatment (Fig. 2E, bottom panels; decreased abundance after antibiotics). These results suggest that the gut bacterial microbiome is a significant determinant of MHV-68 induced disease progression.

### Serp-1 and S-7 peptide treatment efficacy is dependent on the gut bacterial microbiota

We next investigated the potential for dependence of Serp-1 and S-7 therapeutic efficacy on the gut bacterial microbiome in MHV-68 infected IFNγR^−/−^ mice. Mice were treated with medicated drinking water (Table 1) for 10 days, placed on normal water for 24 hours, then infected with MHV-68 and concurrently treated for 30 days with either Serp-1 or S-7 by IP injection. Mice were followed for survival or 3-day follow-up (Fig. 2A).

As previously reported, Serp-1 and S-7 peptide improved survival with 60% of mice surviving to 150 days after MHV-68 infection. In contrast, antibiotic-treated mice had accelerated disease, with a median survival of 19 (Serp-1; *p* = 0.0025; Fig. 1B) and 18 days (S-7; *p* = 0.0339; Fig. 1B). Serp-1 or S-7 efficacy after antibiotic treatment is not significantly different from saline treatment after antibiotics (Serp-1 + Abx vs Saline + Abx, *p* = 0.2012; S-7 + Abx vs Saline + Abx, *p* = 0.9164). Thus, the efficacy of Serp-1 or S-7 treatment and protection in lethal MHV-68 infections was dependent on the gut bacterial microbiome.

### Microbiome analysis identifies candidate taxa associated with Serp-1 and S-7 treatments

**Figure 3:**
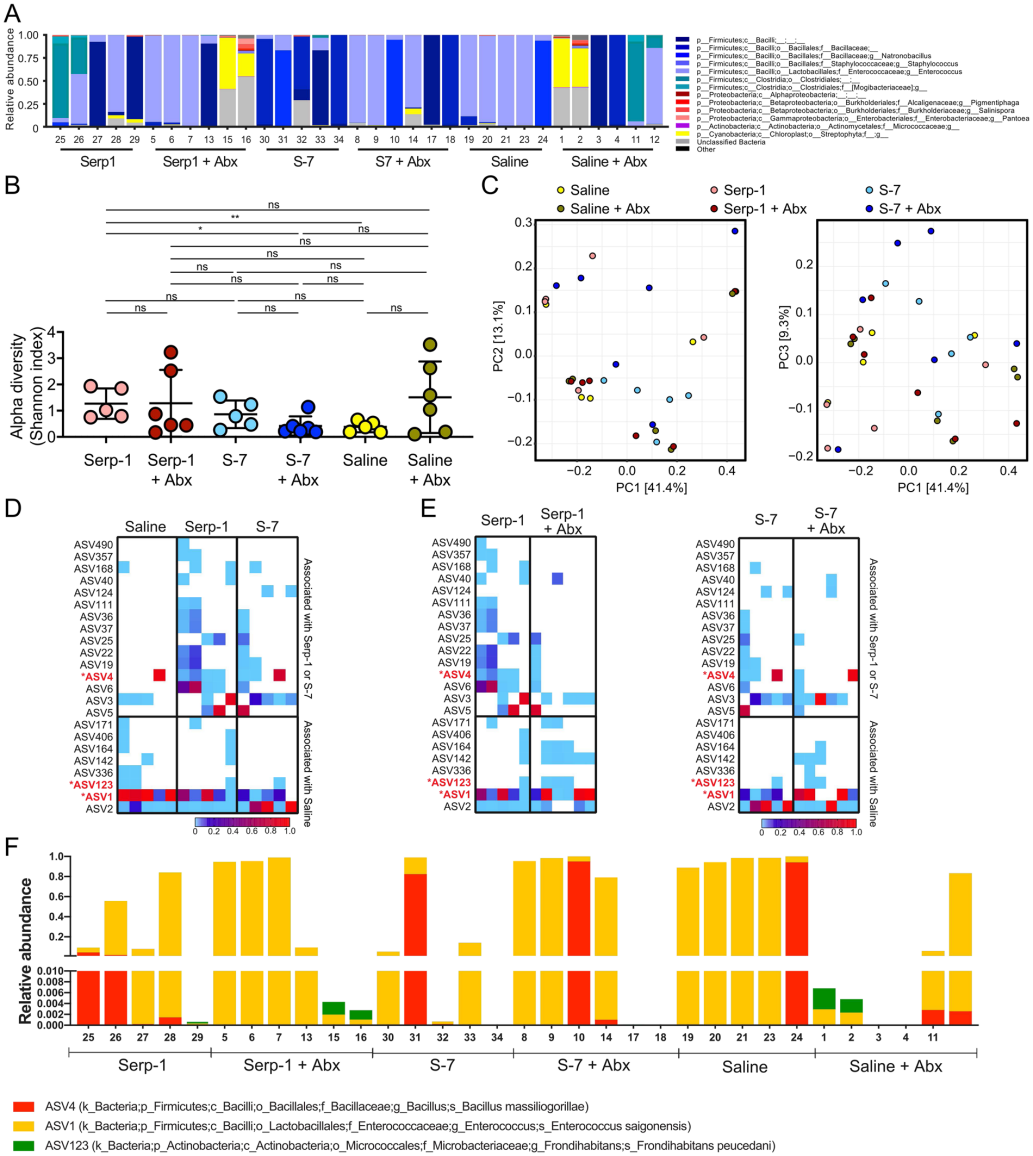
16 S microbiome analysis. (**A**) 16S microbiome relative abundance (genus level) is shown. (**B**) Bacterial alpha diversity (Shannon Index) is shown. Statistical significance was assessed by Mann-Whitney test. (**C**) Principal coordinate analysis (PCoA) plot of weighted UniFrac distances. (**D**) Heatmap showing ASV abundance in mice treated with saline, Serp-1 or S7, in the absence of antibiotics. ASV shown were identified by differential analyses to be associated with Serp-1 or S7 (top section), or saline (bottom section). (**E**) Heatmaps of differential ASV from (**D**) are compared in the absence or presence of antibiotics for Serp-1 treated mice (left) or S-7 treated mice (right). (**F**) Relative abundance of ASV4 (identified as differentially associated with Serp-1 and S-7 treatment), and ASV1 and 123 (identified as differentially associated with saline) in individual mouse microbiomes. ASV taxonomic classification of the most closely related bacterial taxa are shown. Saline + Abx, N = 6; Saline No Abx, N = 6; Serp-1 + Abx, N = 6; Serp-1 No Abx, N = 5; S7 + Abx, N = 6; S7 No Abx, N = 5.

Based on the findings that (1) antibiotic-treated mice accelerated and exacerbated MHV-68 disease, reducing survival time and increasing mortality, and that (2) immune protection conferred by Serp-1 and S-7 was highly sensitive to antibiotic treatment, we profiled the gut bacterial microbiota of mice treated with Serp-1 or S-7. 16S rRNA gene amplicon sequencing was performed on DNA extracts derived from the large intestines of individual mice 3 days post-infection with MHV-68 and after treatment with Serp-1 or S-7 (Fig. 3). Serp-1 and S-7 treated mice, in the absence of antibiotics, had a higher diversity of microbiota (median = 1.270, *p* = 0.0079; median = 0.862, *p* = 0.1508 respectively) than saline treated mice (median = 0.385) (Fig. 3B). Principal coordinate analyses indicate that although the gut bacterial microbiomes of Serp-1 and S-7 treated mice differed from saline treated mice, there was variability within Serp-1 and S-7 treated mice (Fig. 3C, Supplementary Fig. 1B). This finding was consistent with the phenotypic variability observed *in vivo*. Specifically, there was partial protection by Serp-1 and S-7 treatment with 60% survival (Fig. 1B).

We hypothesized that responsiveness to Serp-1 and S-7 treatment may be driven by the presence of “protective” ASVs (differentially associated with Serp-1 and/or S-7 treatment) or the absence of “potentiator” ASVs (differentially associated with saline treatment). Therefore, to identify potential microbe(s) responsible for the microbiome-mediated protection by Serp-1 and S-7, we analyzed the gut bacterial microbiota for discriminant ASVs that were differentially represented in Serp-1 and/or S-7 treated mice compared to saline treated mice, in the absence of antibiotics (Supplementary Fig. 1C–E). Of the 15 ASVs identified as differentially associated with Serp-1/peptide treatments, ASV4, was consistently associated with both Serp-1 and S-7 treatment (Fig. 3D). ASV4 was most closely-related to the sequence of *Bacillus massiliogorillae*^30^. Conversely, ASV1 (most closely-related to *Frondihabitans peucedani*^31^) and ASV123 (most closely-related to *Enterococcus saigonensis*^32^) were identified from 8 ASV candidates as being associated with saline treatment when compared to both Serp-1 or S-7 treatments. Based upon the fact that Serp-1 and S-7 protected mice in an antibiotic-dependent manner, we reasoned that the relative abundance of the discriminant ASVs would be altered in mice that were pre-treated with antibiotics. As predicted, the relative abundance of Serp-1- or S-7-associated ASVs was decreased in antibiotic pre-treated mice, albeit to a more moderate extent in S-7 (Fig. 3E,F, compare “No Abx” to “ + Abx”). These results suggest that the interplay between with bacterial microbiota like ASV4, that are associated with a protective phenotype, and microbiota such as ASV1 and ASV123 that are associated with a patho-exacerbative phenotype, can influence the outcome of immune modulating treatments. In summary, responsiveness to immune modulating therapy in MHV-68 induced disease is associated with specific alterations in the gut bacterial microbiota.

### Antibiotic-dependent exacerbation of early lung pathology of MHV-68 induced disease

**Figure 4:**
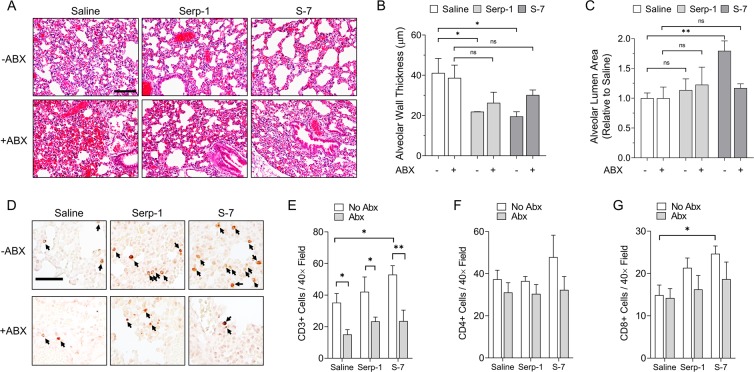
Microbiome-dependent Serp-1 and S-7 efficacy is associated with pulmonary inflammation and increased occupancy of CD3+ and CD8+ cells (**A**) Representative H&E sections of mouse lungs at 3 days follow-up after MHV-68 infection without antibiotic pre-treatment (top row) or after antibiotic pre-treatment (bottom row) and with saline, Serp-1 or S-7 treatment. Scale bar is 100 µm. (**B**) Alveolar wall thickness and (**C**) Alveolar lumen area of mouse lungs at 3 days follow-up as in (**A**). (**D**) Representative CD3 IHC micrographs of mouse lungs at 3 days follow-up after MHV-68 infection without antibiotic pre-treatment (top row) or after antibiotic pre-treatment (bottom row) and with saline, Serp-1 or S-7 treatment. Scale bar is 20 µm. (**E**) Quantification of CD3+, CD4+ and CD8+ cells per 40× field from 3–6 fields per mouse. Statistics in panels B, C and E performed by Two-Way ANOVA with Fisher’s LSD post-hoc analysis. *p < 0.05, **p < 0.01, ***p < 0.001; n.s. is not significant. N = 3 for all conditions.

MHV-68 persistent infection^33^ leads to severe hemorrhagic and consolidating pulmonary pathology^34–36^. Hence, we investigated whether the pulmonary pathology induced by MHV-68 infection reflected the antibiotic-dependent exacerbation we observed in survival analysis. We performed quantitative morphometric analyses of lungs at an early, 3-day follow-up after infection. In the absence of antibiotics, considerable early pulmonary consolidation and inflammation was observed in MHV-68-infected lung tissue in IFNγR^−/−^ mice treated with control saline alone. This severe pulmonary pathology was considerably reduced by Serp-1 or S-7 peptide treatment (Fig. 4A)^10^. Early lung pathology was markedly worse after antibiotic treatment in all conditions (saline, Serp-1 or S-7), with most animals in the saline group also showing hemorrhage in affected regions.

Previous reports have demonstrated that thickening of the alveolar wall/septa and reduction of alveolar lumen area are reliable indicators of pulmonary inflammation in acute laboratory models^37–39^. In this study, quantitative morphometry of the lungs revealed a significant reduction in alveolar wall thickness in MHV-68-infected mice treated with Serp-1 or S-7 versus saline-treated controls that was lost after treatment with antibiotics (Fig. 4B). Without antibiotic pre-treatment and when compared to saline-treated controls, alveolar lumen area was significantly increased in mice treated with S-7 (*p* = 0.0067) with a trend toward an increase in mice treated with Serp-1. Antibiotic treatment nullified any level of protection by treatment with S-7 (Fig. 4C). No diagnostic pathology nor significant changes in inflammatory cell infiltrates on histological analysis was noted in the gut or aorta at this early, 3-day follow-up time (not shown). These results indicate that immune protection against pulmonary inflammation and early stage hemorrhage promoted by Serp-1 and S-7 treatment are microbiome dependent.

### Serp-1 and S-7 promote an increased gut microbiome-dependent CD3+ occupancy in the lungs

T-cell responses play a crucial role in limiting active infection and have been reported as central mediators for managing chronic MHV-68 infection^40–42^. Hence, we examined early, acute phase CD3+ T-cell recruitment to the lungs in MHV-68 infections. Antibiotic treatment significantly reduced CD3+ cells in the lungs of saline-treated mice (Fig. 4E, *p* = 0.0155). Further, S-7 significantly increased, and Serp-1 trended towards increasing, the detectable CD3+ cells in the lungs of infected mice (Fig. 4D,E), indicating that the serpin-mediated protection against MHV-68 induced disease is closely associated with increased pulmonary T-cell activity. Further staining indicates no appreciable effect of Serp-1 and S-7 on pulmonary CD4+ cells (Fig. 4F), while CD8+ staining showed significant increases with S-7 (*p* = 0.0312) and a strong trend towards increase with Serp-1 (*p* = 0.1734) treatment (Fig. 4G). Remarkably, antibiotic treatment that depleted the gut microbiome led to a loss of this observed CD3 infiltration-promoting effects and a loss of CD8 bias of both Serp-1 and S-7 (Fig. 4E-G). Taken together, these results suggest protection from MHV-68 induced disease via CD3 recruitment and surveillance in the lungs, with a CD8 bias, enhanced by Serp-1- or S-7, proceeds via a microbiome-dependent mechanism.

### Serp-1 and S-7 reduce pulmonary MHV-68 levels in a gut bacterial microbiome-dependent manner

**Figure 5:**
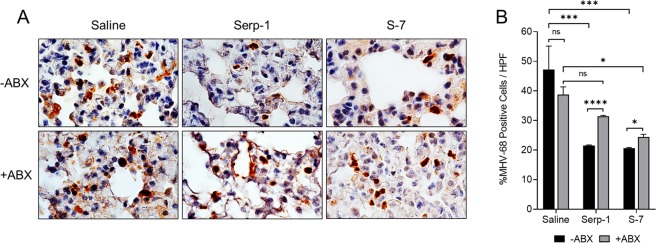
Serp-1 and S-7 reduce MHV-68 presence in a gut bacterial microbiome-dependent manner (**A**) Representative IHC images of 100 × fields depicting MHV-68 antigen staining in the lungs of mice at 3 days follow-up after MHV-68 infection without antibiotic pre-treatment (top row) or after antibiotic pre-treatment (bottom row) and with saline, Serp-1 or S-7 treatment. (**B**) Quantification of percent of MHV-68 positively stained cells per 100 × field from 3 fields per mouse. N = 3 for all conditions.

Because T-cells are crucial for control of MHV-68 infection, we hypothesized that the promotion of CD3 T-cell infiltration would lead to reduced viral levels in the lungs. Antibiotic treatment did not change the amount of MHV-68 antigen staining in the lungs of mice treated with saline control alone (Fig. 5A,B). However, Serp-1 and S-7 significantly reduced MHV-68 antigen levels in the lungs in mice without antibiotic treatment (Fig. 5B; Serp-1, *p* = 0.0009; S-7, *p* = 0.0003). These decreases in detectable MHV68 were partially reversed after antibiotic pre-treatment in MHV-68-infected mice (Figs. 5 and S3). Thus, Serp-1 and S-7 initiate a gut bacterial microbiome-dependent reduction in MHV-68 levels in the lungs.

## Discussion

Compelling evidence has established an important role for the gut microbiome in immune responsiveness^43–45^ as well as the pathogenesis of various diseases, including those with primary involvement outside of the gut, such as respiratory^46^, hepatic^47^, renal^48^, neurologic^49^, autoimmune^50^, rheumatoid^51^ and cardiovascular^52–54^ conditions. Some research groups have demonstrated that targeted manipulation of the gut microbiome to correct dysbiosis, as for fecal transplants, may aid in treatment of certain diseases. For example, a small, randomized clinical trial (NCT02636647) found improved outcomes in recurrent hepatic encephalopathy patients treated by fecal microbiota transplant from a single, rationally selected donor enriched with Lachnospiraceae and Ruminococcaceae (taxa determined to be depleted in hepatic encephalopathy patients)^55^. A second trial (NTR1776) found that fecal microbiota transfer from lean donors to obese donors partially reversed symptoms of metabolic syndrome, including improvements in insulin resistance^56^. Aside from correcting dysbiosis, recent evidence supports the hypothesis that microbiome composition also plays a significant role in determining the efficacy of some treatments. It was recently found that low gut microbiome levels of *Akkermansia muciniphila* caused non-responsiveness in patients receiving PD-1 blockade immunotherapy for epithelial tumors^57–59^, reinforcing the importance of the microbiome in treatment efficacy, as highlighted by a critical earlier study that identified a positive correlation between *Bacteroides fragilis* and anti-CTLA4 therapy efficacy for melanoma^60^. Alternatively, some treatments may inadvertently alter the gut microbiome and predispose to increased pathogenic infections. For example, it was recently demonstrated that proton pump inhibitors significantly reduced microbial diversity with changes in more than 20% of bacterial taxa, leading to increases in *Enterococcus*, *Streptococcus*, *Staphylococcus* and the potentially pathogenic *Escherichia coli* species^61^. Thus, an understanding of how the gut microbiome influences, and is influenced by, new therapeutic treatments is crucial.

Trans-kingdom interactions may also play a crucial role in dictating disease pathogenesis in mammalian hosts. For viruses which require enteric bacteria for replication, antibiotics may reduce viral load. Numerous studies have demonstrated this viral-bacterial interaction in laboratory systems, such as murine and human norovirus infection^62,63^. For other viruses, however, loss of the bacterial microbiome may lead to worsening clinical disease. A recent study by Thackray *et al*. reports that oral antibiotic (ampicillin) treatment exacerbates disease severity in flavivirus infections by modulating flavivirus-specific CD8 responses^64^, suggesting a microbiome, immune system and virus interaction. These disparate observations, which depend substantially on (a) the virus being studied, (b) the host system and (c) the antibiotic regimen, highlight how the virome is still a poorly understood, and consequently under-appreciated, component of health and disease^65^.

We recently reported a severe gastrointestinal pathology in a significant fraction of IFNγR^−/−^ mice intraperitoneally infected with MHV-68^11^, a model for systemic arteritis and hemorrhagic pneumonia^9,10^. In this same model, we also previously reported therapeutic efficacy with the MYXV-derived serine protease inhibitor Serp-1^10^ as well as peptides derived from the Serp-1 reactive center loop^22,25^. On this basis, we undertook a systematic analysis of the effect of Serp-1 and the RCL-derived Serp-1 peptide S-7 on MHV-68 induced disease.

A broad-spectrum antibiotic cocktail was used to suppress the gut microbiome prior to MHV-68 infection. We found that suppression of the gut microbiota prior to MHV-68 infection accelerated disease, with increased lethality and earlier death occurring at approximately twice the rate of untreated mice (Fig. 2C). This acceleration of MHV-68 disease was accompanied by a dramatic post-antibiotic dysbiosis and a suite of candidate ASVs with increased or decreased relative abundance associated with worsened disease outcomes.

Furthermore, we demonstrate here that bacterial microbiome interactions were required for a protective immune response induced by Serp-1 or S-7^10^. Microbiome ablation completely abolished treatment efficacy and improved survival (Fig. 1B). Probing further, we found that Serp-1 and S-7 facilitated protection against lung inflammation and consolidation associated with an increase in CD3+ cell invasion (with a CD8+ bias) and a reduction of MHV-68 staining in the lungs (Figs. 4 and 5). This finding is in agreement with previous reports that T-cell surveillance (effected by CD8+ cells) is critical for limiting acute GHV infection and that a progressive loss of T-cells is associated with worsened GHV disease^40–42^. The loss of Serp-1-based treatment efficacy was not expected to be due to direct interactions between the antibiotics and immune modulating treatments for a number of reasons: (1) the antibiotics used in this study have a relatively short half-life (1–7 hours) and previous studies report negligible concentrations 24 hours after dosing^66–69^ – a timeline we incorporated in this study design; (2) we observed a rapid recovery of microbial burden within three days (Fig. 2B); (3) the antibiotics in our cocktail are not known to suppress immune responses on their own, and in some reports are observed to enhance T-cell activity^70^; (4) it is not expected that the loss of activity is due to direct interactions with the antibiotics because *in vitro* inhibition of uPA activity by Serp-1 is unaffected by the presence of the same antibiotic cocktail (Fig. S2). A recent report by Yang *et al*. indicated that in the short-term, antibiotics can induce metabolite changes which have systemic, suppressant effects on immune function in the peritoneal space by altering respiratory activity^44^. Yang *et al*., however, utilized an *E. coli* infection and the antibiotic, ciprofloxacin, was given within 4 hours of infection and maintained during the course of the infection. Here, we utilize a gammaherpesvirus infection with longer pathogenic kinetics and remove antibiotics 24 hours prior to infection, wherein any trace of ciprofloxacin would be undetectable^66^, and we are not aware of any other reports suggesting immune suppressant effects of the other antibiotics in our cocktail. Taken together, these data provide the first evidence that microbiome interactions are essential for a protective immune response, induced here by Serp-1 and S-7 treatment. We have previously found Serp-1 to function through the urokinase-type plasminogen activator receptor (uPAR) in systems involving immune cell infiltration (e.g., wound healing^71^, chemokine-induced ascites^72^). Levels of uPAR have been found to increase substantially in incidences of infection and sepsis^73^. Thus, future work will investigate the possibility that the microbiome is modulating levels of binding partners essential for Serp-1 and S-7 function in MHV-68 infection.

**Figure 6:**
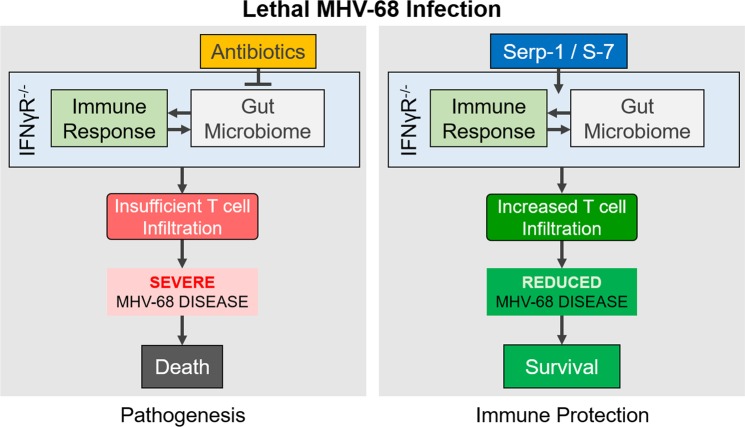
Proposed model (**Left**) In uninterrupted lethal MHV-68 infection of IFNγR^−/−^ mice, the gut microbiome and immune response interact to mount an ultimately insufficient response of cells such as T-cells to affected tissues (e.g., the lungs), leading to severe disease and death. Antibiotics suppresses the immune stimulatory effects of the bacterial microbiome, reducing further the immune response and worsening the disease. (**Right**) In immune modulator-mediated protection by treatments such as Serp-1 or S-7, the interactions leading to a sufficiently mounted immune response are enhanced, promoting an increased T-cell infiltration to affected tissues, reducing disease pathology and leading to survival.

We propose here that transkingdom interactions with the gut bacterial microbiome are required for host innate immunity to mount a protective response against lethal gammaherpesviral disease (Fig. 6). When MHV-68 infection causes viral pulmonary inflammation and sepsis, as induced in the MHV-68 infection model, host innate immune responses mediated by gut bacterial microbiota interactions leads to the recruitment of immune cells (e.g. T-cells) to the lungs. However, antibiotic ablation of the gut bacterial microbiota alters pathologic outcome by preventing effective immune response responses leading to acceleration of MHV-68 inflammatory pathogenesis. Immunomodulatory treatments (like Serp-1 and S-7) can induce a stronger innate immune response (such as T-cell recruitment) through (other) bacterial microbiota interactions. Thus, outcomes of GHV infection are determined by the interplay of host innate immunity and microbiota interactions.

Interestingly, the gut microbiota of all Serp-1 and S-7 treated mice did not clearly cluster separately from saline treated mice. Thus, the dynamics of selected increased or decreased microbiome ASVs is associated with worsened pathology or alternatively protective immune modulation. Given that not all mice were fully protected by Serpin treatment, we speculate that the gut microbiota heterogeneity of Serp-1- or peptide-treated mice may reflect the true variability in immunological outcomes (i.e. 60% protection). We found specific ASVs that were either increased in response to Serp-1/peptide treatment (e.g. ASV4), or decreased when compared to saline treatment (e.g. ASV1 and ASV123).

Although the current study design could not assess the predictive value of these discriminant ASVs on individual mice survival outcomes, future studies will need to be performed in order to stratify the role of different microbiome sub-populations by specific (i.e., single) antibiotic treatment as well as defined microbial reconstitution (e.g., with the Altered Shaedler Flora community or similar)^74^. Taken together, these findings suggest that the responsiveness to immune modulations in MHV-68 disease involves a combination of interactions with protective microbiota and patho-exacerbative microbiota. Further work will be needed to establish a direct role for bacteria related to these select ASVs to prove cause-and-effect relationships and to define a precise mechanism of bacterial microbiome influence on immune responses in GHV disease.

With this study, we report that the pathogenicity of MHV-68 infection and effective treatment with the immune modulating serpin, Serp-1, and serpin peptide, S-7, is substantially accelerated by suppression of the gut bacterial microbiome with antibiotics. MHV-68 is a model virus for studying GHV pathogenesis and severe viral sepsis^4,75^, therefore this finding may have implications for the pathogenesis and treatments of other GHV, such as KSHV and EBV, where adverse responses to antibiotics are clinically recognized^76^. We further report here the modulation of gut microbiome composition by an immune modulating protein and peptide. In conclusion, (1) microbiome changes may have significant impact on therapeutic immune modulating proteins^77,78^ and peptides^79,80^, and (2), antibiotic treatment may increase severity of GHV infections.

## Methods

### Ethics statement

All animal studies conform to local and national guidelines for animal care and experimentation. Protocols were approved by the local Institutional Animal Care and Use Committee (IACUC) of University of Florida (#201604234_01).

### Animals

IFNγR^−/−^ mice (B6.129S7-*Ifngr1*^*tm1Agt*^/J) were purchased from the Jackson Laboratory (Bar Harbor, ME, USA). Animals were housed in barrier conditions at the University of Florida Animal Care Services vivarium and bred under specific pathogen-free conditions. Mice were weaned at 3 weeks, maintained on a 12-hour light-dark cycle and were fed water and standard rodent chow *ad libitum*.

### Antibiotic treatment

At 4 weeks of age, IFNγR^−/−^ mouse cohorts were transferred from the ABSL1 colony to a separate, ABSL2 colony. Gut microbiome suppression was achieved by replacing standard drinking water with autoclaved reverse osmosis water (obtained from the same animal care facility) containing an antibiotic cocktail (Table 1) composed of Streptomycin (2 g/L), Gentamicin (0.5 g/L), Bacitracin (1 g/L) and Ciprofloxacin (0.125 g/L) for 10 days. One day (24 hours) prior to infection, medicated water was replaced with standard animal care facility water, which was maintained for the remainder of the experiment.

### MHV-68 infection

On day 11 (after 10 days of antibiotics) mice (5 weeks old) were infected with MHV-68 at a dose of 12.5 × 10^6^ PFU in 0.1 mL DMEM by intraperitoneal (IP) injection as previously described^22,25,27,29^. Mice were returned to the colony and monitored for signs of distress for the duration of the experiment. Mice were either followed for 150 days to determine survival or euthanized at 3 days post-infection and organs harvested into formalin for histology or RNA*later* (Thermo Scientific, USA) for microbiome analyses. Details on the numbers of MHV-68-infected mice in this study are detailed in Table 2.

**Table 2:** MHV-68-infected mice in this study.

Treatment	Antibiotics (ABX)	Follow-up	# of mice
Saline	No	3 days	6
	Yes	3 days	6
	No	150 days	12
	Yes	150 days	5
Serp-1	No	3 days	6
	Yes	3 days	6
	No	150 days	5
	Yes	150 days	5
S-7	No	3 days	6
	Yes	3 days	6
	No	150 days	5
	Yes	150 days	5
	**Total # Infected Mice**		**73**

### Gut bacterial microbiome sample processing

Large intestine samples preserved in RNA*later* were processed for total genomic DNA isolation using the ZymoBiomics® miniprep kit (Zymo Research) according to manufacturer’s recommended procedure. Isolated DNA was quantified using a NanoDrop 2000C (Thermo Scientific) and stored at −80 °C. Samples were analyzed in the Arizona State University KED Genomics Core for whole-sample 16 S rRNA gene amplicon sequencing. DNA library preparation for Illumina® MiSeq platform was prepared according to the protocol from Earth Microbiome Project (http://www.earthmicrobiome.org/emp-standard-protocols/16s). The 16 S primer set 515f-806r^81^ was used for 2 × 150 pair-ended sequencing.

### 16S rRNA gene amplicon sequencing and analysis

Illumina MiSeq sequencing reads (2 × 150 bp) of the 16 S rRNA gene V4 region^82^ were analyzed with QIIME 2 (ver 2017.12)^83^ for 34 samples: Saline + Abx (n = 6), Saline No Abx (n = 6), Serp-1 + Abx (n = 6), Serp-1 No Abx (n = 5), S7 + Abx (n = 6), S7 No Abx (n = 5). Sequencing reads were processed with DADA2 to infer Amplicon Sequence Variants (ASVs) at a 97% identity threshold using Greengenes database (version 13.8)^84^. To account for inter-sample depth variability, all samples were rarefied to 55,000 reads per sample (10 iterations) (Fig. S1A). One sample (12,107 reads; Saline No Abx group) was omitted due to insufficient reads and the remaining 33 samples were preserved for further analysis. ASV richness, alpha diversity (Shannon’s Diversity Index) and beta diversity (UniFrac distance) were calculated using QIIME 2. Statistical analyses (read depth, ASV richness and Shannon Index comparisons) were performed with Mann-Whitney test. Bars are median ± standard error. P-value less than 0.05 was considered significant. *p < 0.05, **p < 0.01, ***p < 0.001, ****p < 0.0001; n.s. is not significant. PCoA was performed with weighted unifrac distance in QIIME 2. To identify discriminating ASVs associated with the respective treatments, LEfSe (Linear discriminant analysis Effect Size)^85^, DESeq. 2 (version 1.20.0)^86^ and likelihood ratio tests were performed in R studio (Version 1.1.456) (Fig. S1C–E). Discriminating analyses were first performed compared Serp-1 (no Abx) to saline (no Abx) mice, S-7 (no Abx) to saline (no Abx) mice, and finally as a combined group of Serpin-treated mice [Serp-1 (no Abx) and S-7 (no Abx)] to saline (no Abx mice). Discriminant ASVs identified in these analyses were pooled and validated in heatmap and abundance analyses (Fig. 4D–F).

### Histopathology

Mice were euthanized at 3 days follow-up after infection by carbon dioxide asphyxiation followed by cervical dislocation. Organs were harvested and fixed in 10% neutral buffered formalin. Samples were dehydrated through graded alcohol, paraffin-embedded, sectioned into 4–6 µm ribbons, captured on charged glass and dried overnight at 37 °C prior to processing for histopathology. Slides were rehydrated, stained with Gill’s hematoxylin No.3 and Eosin Y (H&E) according to standard procedure, dehydrated and mounted in Cytoseal XYL (Thermo Fisher Scientific, USA).

### Quantitative morphometry

H&E-stained aorta, lung and colon sections were imaged with a 20 × /0.5NA objective on an Olympus BX51 microscope equipped with an Olympus DP74 camera operated by cellSens Dimensions v1.16 software. Objective-calibrated measurements of alveolar septal thickness and lumen area were collected using cellSens Dimensions. At least 50 measurements were collected and averaged for each mouse, and at least three mice were examined for each group. Images were processed for visualization in figures using ImageJ/FIJI v1.52i^87^.

### Immunohistochemistry

FFPE blocks containing lung tissue were sectioned into 4–6 µm ribbons, captured on charged glass and dried overnight at 37 °C prior to processing. Slides were rehydrated and epitopes retrieved by boiling in sodium citrated buffer. Endogenous peroxidases were quenched with 3% hydrogen peroxide, slides were blocked in 5% bovine serum albumin in TBS/0.1% Tween 20 and sections were probed with rabbit polyclonal antibody against CD3 (Abcam ab5690; 1:200), CD4 (Abcam ab183685; 1:1000) or CD8 (Abcam ab209775; 1:2000). For MHV-68 detection, 1:500 rabbit anti-serum or pre-immune serum (as control) were used for immunostaining (kind gift of Dr. H.W. Virgin III^9^). 1:500 goat anti-rabbit HRP was used for secondary staining (Jackson Immuno Research 111-035-144). Antigens were revealed with ImmPACT DAB (Vector Labs, USA) and mounted with Cytoseal XYL. Sections were imaged with a 40 × /0.75NA objective.

### Viral load determination by qPCR

Total DNA was isolated from each FFPE sample (4 × 5 µm thick sections for each sample was used) using the QIAamp DNA FFPE Tissue Kit according to the manufacturer’s instructions. Samples were quantified using a DS-11 series spectrophotometer/fluorometer (Denovix, USA). Quantitative PCR (qPCR) was undertaken to investigate relative viral load using the following reaction per sample: 10 µL SsoAdvanced Universal SYBR Green Supermix (Bio-rad Laboratories Inc., USA), 0.4 µl each of the primers 65 F (5′-GTCAGGGCCCAGTCCGTA-3′) and 65 R (5′-TGGCCCTCTACCTTCTGTTGA-3′), 200 ng of DNA and water up to 20 µL total volume. Reactions were run in triplicate with controls, and a standard curve using pCR2.1 Topo plasmid (Thermo Fisher Scientific, USA) containing the cloned target MHV region^35^. The following cycling conditions used on a CFX96 (Bio-rad Laboratories, Inc., USA) instrument: 95 °C for 20 sec, 40 cycles (95 °C for 15 sec, 60 °C for 20 sec), followed by a melt curve analysis.

### Statistical analysis

Survival and pathology statistics were analyzed with GraphPad Prism v8.0.1. Kaplan-Meier survival statistics were calculated using Log-rank (Mantel-Cox) testing. For visualization, individual comparison curves are presented. Lung pathology statistics were compared using a Two-Way ANOVA with a Fisher’s LSD or Tukey’s post-hoc test. Bars are mean ± standard error. P-value less than 0.05 was considered significant. *p < 0.05, **p < 0.01, ***p < 0.001, ****p < 0.0001; *n.s*. is not significant.

## Supplementary information

Supplemental Information.

## Data Availability

Sequence data has been deposited to the NCBI Sequence Read Archive under BioProject accession number PRJNA517927.
