# Supplementary material for: Immune protection is dependent on the gut microbiome in a lethal mouse gammaherpesviral infection
Source: Sci Rep. 2020 Feb 11;10:2371. doi: 10.1038/s41598-020-59269-9 (PMC7012916; doi:10.1038/s41598-020-59269-9)
Supplement: Supplementary file 1 — Supplemental Information. [file 41598_2020_59269_MOESM1_ESM.docx]

**SUPPLEMENTAL INFORMATION**

**Immune protection is dependent on the gut microbiome in a lethal mouse gammaherpesviral infection**

Jordan R. Yaron^1,2,†^, Sriram Ambadapadi^1,2,†^, Liqiang Zhang^1,2^, Ramani N. Chavan^3^, Scott A. Tibbetts^4^, Shahar Keinan^5^, Arvind Varsani^3,6,7,8^, Juan Maldonado^3,9^, Simona Kraberger^2,3^, Amanda M. Tafoya^1,2^, Whitney L. Bullard^4^, Jacquelyn Kilbourne^1,2,3^, Alison Stern-Harbutte^4^, Rosa Krajmalnik-Brown^3,10,11^, Barbara H. Munk^1^, Erling O. Koppang^12^, Efrem S. Lim^3,*^, Alexandra R. Lucas^1,2,4,*^

*^1^Center for Personalized Diagnostics, The Biodesign Institute, Arizona State University, Tempe, Arizona, USA*

*^2^Center for Immunotherapy, Vaccines and Virotherapy, The Biodesign Institute, Arizona State University, Tempe, Arizona, USA*

*^3^Center for Fundamental and Applied Microbiomics, The Biodesign Institute, Arizona State University, Tempe, Arizona, USA*

*^4^Department of Molecular Genetics & Microbiology, College of Medicine, University of Florida, Gainesville, Florida, USA*

*^5^Cloud Pharmaceuticals, Research Triangle Park (RTP), North Carolina, USA*

*^6^School of Life Sciences, Arizona State University, Tempe, Arizona, USA*

*^7^Center of Evolution and Medicine Arizona State University, Tempe, Arizona, USA*

*^8^Structural Biology Research Unit, Department of Integrative Biomedical Sciences, University of Cape Town, Rondebosch, Cape Town, South Africa*

*^9^KED Genomics Core, Arizona State University, Tempe, Arizona, USA*

*^10^Swette Center for Environmental Biotechnology, The Biodesign Institute, Arizona State University, Tempe, Arizona, USA*

*^11^School of Sustainable Engineering and the Built Environment, Arizona State University, Tempe, Arizona, USA*

*^12^Department of Basic Sciences and Aquatic Medicine, Faculty of Veterinary Medicine, Norwegian University of Life Sciences, Oslo, Norway*

^†^ to be considered co-first authors

* Correspondence: The Biodesign Institute at Arizona State University, 1001 S. McAllister Ave., Tempe, AZ 85281, USA. E-mail: [arlucas5@asu.edu](mailto:arlucas5@asu.edu); Phone: +1-352-672-2301

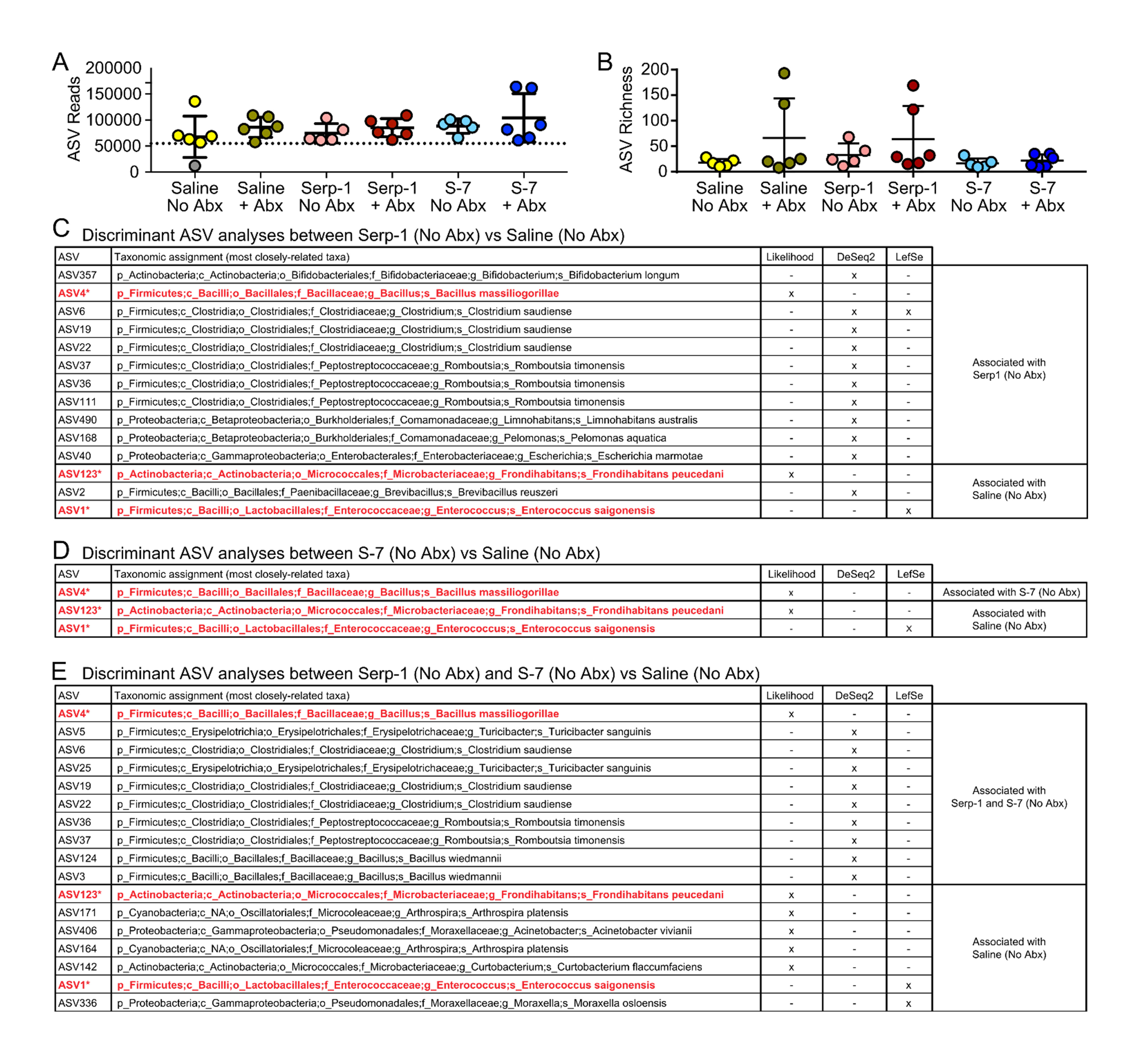


**Figure S1**. (A) ASV reads of each sample is shown. One saline no abx sample was omitted for low reads (12,107 reads), as labeled in gray. There was no statistically significant difference between the groups as assessed by Mann-Whitney test. (B) Richness (number of observed bacterial ASV) is plotted (n =33). (C) Results of differential analyses by likelihood test, DeSeq2 and LefSe between saline no abx mice and Serp-1 no abx mice are shown. (D) Results of differential analyses by likelihood test, DeSeq2 and LefSe between saline no abx mice and S7 no abx mice are shown. (E) Results of differential analyses by likelihood test, DeSeq2 and LefSe between Saline no abx mice and combined Serp1/S7 no abx mice are shown. In (C), (D) and (E), ASV identified as significantly different in their respective analyses are indicated by “x”, ASV not significantly different are indicated by “-”.


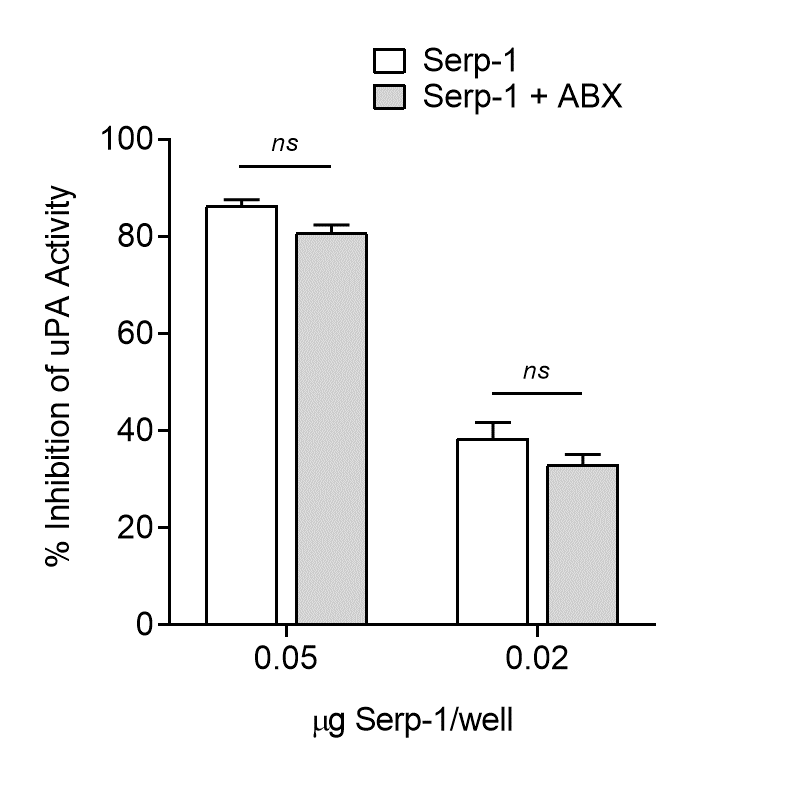


**Figure S2**. Serp-1 inhibition of uPA activity in an *in vitro* substrate conversion assay is not affected by the presence of a broad-spectrum antibiotic cocktail.


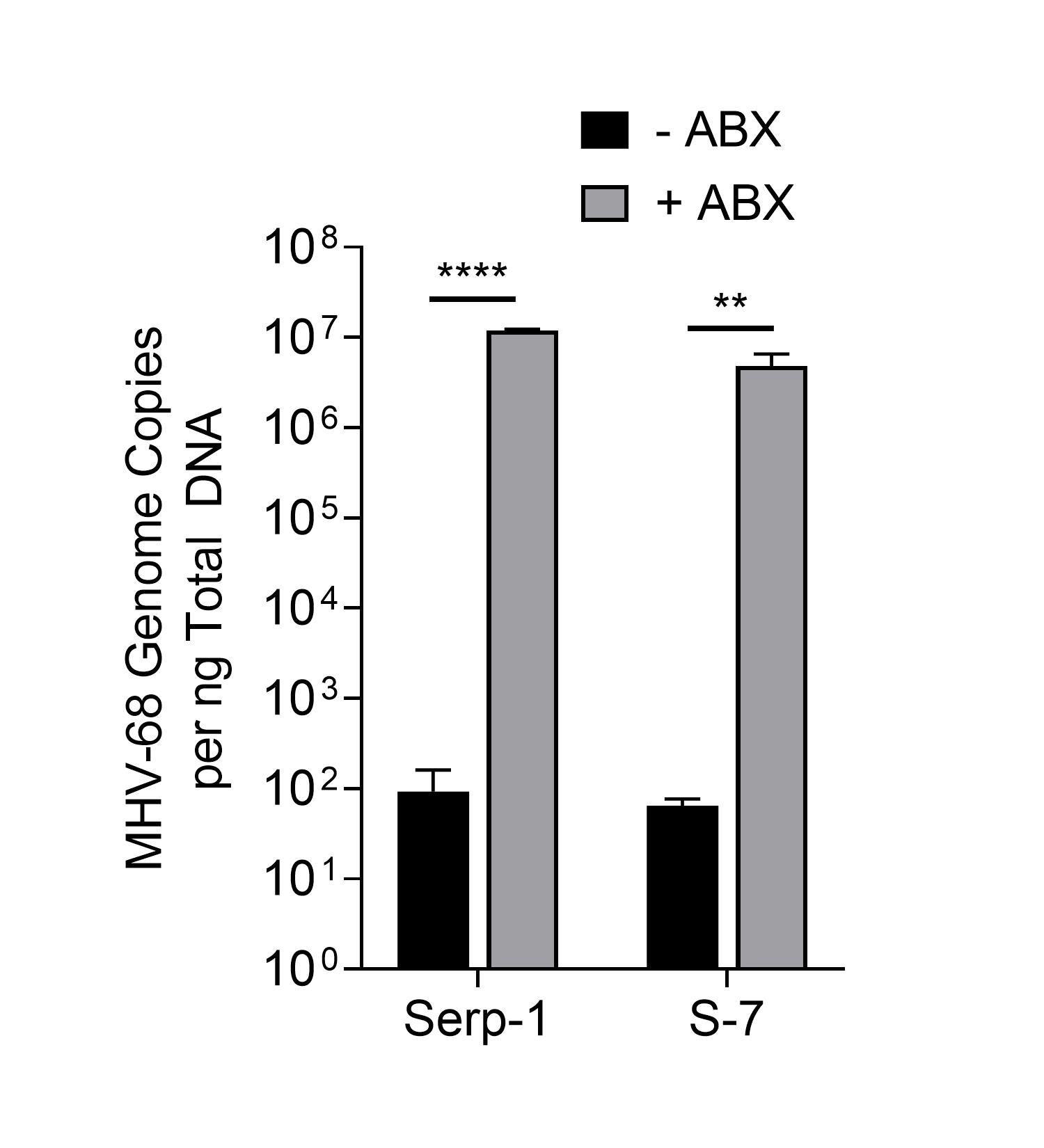


**Figure S3**. Antibiotics increases the viral load of MHV68 in the lungs.
